# The Hospital Nursing Supplement

**Published:** 1893-03-04

**Authors:** 


### "The Hospital" Nursing Mirror.

Being the Extra Nursing Supplement of "The Hospital" Newspaper.

[Contributions for this Supplement should be addressed to the Editor, The Hospital, 428, Strand, London, W.C., and should have the word "Nursing" plainly written in left-hand top corner of the envelope.]

### News from the Nursing World.

### SICK AND POOR.

The East London Nursing Society, now affiliated with Q.V.J.I., has issued an interesting report of last year's work. With such a record of cases, it is pitiable to read of an annual diminution of income. Unless some new donors appear the work must be curtailed. Fresh subscribers are sorely needed, and surely they will come forward to support this valuable and practical good work. The late Matron, Miss Taylor, whose lamented death occurred last autumn, wrote: "Poor as our people are, they are very ready to give what they can for others, and gladly put their pennies in the box that they may send nurse on to some one else who is bad." Miss Taylor's deep personal interest in her workers is also well known. She wrote, shortly before her death: "I am always watchful to get my nurses to put into the Royal National Pension Fund for nurses, towards which the society offers such good help."

### FIXED VERSUS MOVABLE FEASTS.

Some correspondents have got it into their heads that the nurses at certain hospitals suffer in health because their daily luncheon-hour extends from ten to half-past twelve. It is contended that nurses are apt to get enthusiastic over their work, and that such an arrangement often leads to nurses omitting to get any lunch at all. In other words, a movable feast is declared to be unsuited to the circumstances of a hospital nurse. We have made some inquiry into the matter from the authorities of the institutions specially referred to, and we are informed that although the luncheon hour does extend from ten to half-past twelve, as cold meats, cheese, salads, and so forth form the menu, nurses prefer to have an option as to the particular hour at which they shall partake of this meal as it enables them to arrange with their friends on the staff to take it together. Further, it is not the fact that the nurses do not partake of this meal, seeing that the whole of the liberal dietary is very often consumed. We are bound to say that in our view it is better to have a definite hour, and to insist upon the nurses leaving their wards and spending the allotted time in the dining-room, so as to secure that they shall have the maximum of opportunity for partaking of food in sufficient quantity at regular intervals. As the point has been raised, however, we should be glad to hear what view the nurses and officials take of this question.

### HOME MISSIONS.

More work is waiting for women, as we find through Miss Florence Balgarnie's letter to the *Daily News*. This lady appeals for a matron's constant presence in English police-stations, and tells us they are already installed in all large places in the United States. Unhappily there are few centres here where their presence is enforced even to come "when required," as the somewhat vague regulations express it. The subject is brought forward just now by a sad enough incident at Wolverhampton, a sober, paralised woman having been treated as drunk and incapable, death ensuing next day. Surely some middle-aged women, whose experience stands them in lieu of modern "training," might well be employed as guardians and attendants of the unhappy women and girls who, through misfortune or sin, are temporarily "locked up." Such a "misadventure" as the one at Wolverhampton ought never to occur again in Christian England. "In Scotland," Miss Balgarnie writes, "it is far otherwise." Surely what Edinburgh and Glasgow can do is equally possible in towns south of the border.

### PROGRESS.

The annual report of the Kent and Canterbury Institute for Trained Nurses shows several excellent reforms. The nursing staff is increased, and better trained nurses are employed, at higher salaries. Some difficulty was experienced in securing fully qualified nurses, but this will be obviated in the future by employing the probationers, for whom adequate training is secured by the institute at well-known provincial hospitals. The decreased expenditure under Miss Shaw, the Lady Superintendent's able management, was cordially commended by the committee.

### A COTTAGE HOSPITAL.

The fourth annual report of the Weybridge Cottage Hospital and Parish Nurse Fund has recently been laid before the subscribers. In the last year the number of in-patients has been rather lower than usual, and the Matron has been much occupied with the nursing of the sick poor in their own homes. Amongst other gifts received during the year, valuable presents of meat, &c., are noted, and Dr. Graham continues his superintendence of the garden. It is very pleasant to find what cordial local support a well-managed cottage hospital generally secures.

### PRACTICAL CYCLISTS.

The Middlesbrough Nursing Association appears to be meeting with the appreciation it deserves. The Superintendent (Miss Purvis) was thanked at the annual meeting for the unselfish and devoted services of herself and her staff. The latter now consists of two district and two private nurses, and a third district nurse has just been engaged. The committee called attention to the fact of no charge being made for the nurses services, and also made mention of no subscriptions being received from working men. It was therefore proposed to invite employers to allow boxes to be placed at their works, to give those who chiefly benefit by the association an opportunity of adding their offerings to the donations of their masters. The accommodation for the nurses is not altogether satisfactory, but improvements will be made, and more adequate premises secured so soon as the funds of the association permit. The Cyclists Costume and Blossom Parade brought the handsome contribution of £55 19s. 7d., and it is to be hoped that other societies will shortly feel inspired to follow the example of Mr. D. Caldick, who chiefly managed this successful enterprise. In other districts the co-operation of workmen has been so cordial that we hope soon to hear that the Middlesbrough folks are no whit behind their neighbours in giving substantial support to the nurses, whom they appear already highly to value.

### ECCENTRIC INSTRUCTION.

"And now I will tell you a little about the lungs," said a lady lecturer the other day. "They are two bags, one containing bad air and the other one good air. Sometimes one lung gets diseased, and then it is no more use, but people can live quite well with one." The audience listened in puzzled silence, and felt that the belief in good air which had been inculcated by former teachers was rudely dispersed. What could be its value if people got along all right with only one lung, and that possibly the "bag containing the bad air"! Nurses get their knowledge of poultices considerably enlarged in an out-patient department, for they see persons return days afterwards with an application which has been kept on continuously in spite of advice given to the contrary. The facts which such nurses witness are far stranger than fiction, and doubtless it is from pondering on these that experienced women have made such useful attempts to teach the poor in their own homes some of the elements of sick nursing. Home nursing lectures are going on at Banbury just now, and the *Banbury Guardian* reports a somewhat primitive fomentation. The instruction given reads thus: "The flannel is raised by means of a stick, and allowed to drip for a moment before being placed in the inner part of a well-warmed roller towel." This is probably what many people do without teaching, but a lecturer should be herself acquainted with the use of the simple and effectual wringer in which the flannel is placed before, not after its conversion into a fomentation. We also think it unfortunate that the Banbury audience should be advised to pour the water on to the linseed, instead of shaking the meal into the boiling water; the latter makes a poultice, but the former is something of a pudding.

### NURSING IN PARIS.

The Municipal Council recently held a reception for nurses and other hospital officials in the Grand Salon des Arcades of the Hotel de Ville. In the course of his address, M. Santon, the President, said that French people did not keep themselves well informed as to the progress made in other countries, and in the organisation of hospitals they are sometimes rather behind the times.

### NURSING FOR NATIVES.

Fresh fields for nurses continually display themselves, and the *Madras Weekly Mail* now puts in an urgent appeal for the Native Army in India. The authority of Surgeon-Colonel Donnelly is given for some really appalling figures—that of "6,000 followers who entered the portals of the General Hospital only 400 recovered to return to their duties in the field.' Most of these patients suffered "from malarial fever and dysentery." The nursing system in native military hospitals is represented by two ward attendants, whose duty it is to carry out the doctors orders. The kind of individuals selected for this important work is described by the *Madras Weekly Mail*: "Every care is taken by the military authorities that nothing but material that is unfit for a Sepoy should reach the hospital, the men who undertake this work being carefully informed—under regulations—before undertaking their new functions that they must abandon all hope of future promotion. . . . The effect of such a system is either to provide the hospital with superannuated old fossils, or men of less than average physique and brain capacity." The cooking for the sick is also said to be bad, and the soup is described as foul tank water flavoured with a little meat or bones, ghee pepper, and onions. The doctors are most efficient, and are frequently driven to undertake nursing details, in addition to their own duties. An adequate supply of trained nurses for native solders in India would remove the reproach which the same paper utters: "Are we, then, to continue to give less attention to the comforts during sickness 'of men who lay down their lives for their country' than we do for the pauper, who claims in civil hospitals the indulgence of the taxpayer?"

### SHORT ITEMS.

At East Grinstead Hospital 42 cases have been treated during the year, and the new Matron, Miss E. Oxtoby, seems to be as popular as her predecessor, Miss Kate Taylor, the Committee expressing cordial appreciation of the efficiency of both nurses.—St. Lawrence's Catholic Home, Dublin (a branch of Q.V.J.I), reports 123 cases attended by the four nurses during the last three months, and 2,581 visits paid. The nurses have special courses of lectures from surgeons and physicians. —In a paper prepared for the Missionary Conference at Bombay, Mr. Wellesley Bailey, Secretary and Superintendent of the Mission to the Lepers, says that the numbers of persons suffering from this disease in India alone is variously estimated at from 100,000 to 500,000, and he believes the latter number to be within the mark! He also speaks of a present need for a Home under medical superintendence and trained nursing for Anglo-Indians suffering from leprosy.—The Matron of the Perth Small-pox Hospital has the small-pox, although she was thought to be protected by a previous attack. —At Leicester all the unvaccinated nurses have been attacked by small-pox.—It is under discussion to have a Floating Hospital ready for cholera patients on the Thames, between Blackfriars and London Bridge.

### THE DISABLED NURSE.

The disabled nurse has again written gratefully in acknowledgment of the kind interest shown by the readers of The Hospital. In answer to a question from one subscriber, the invalid expresses regret that she cannot manage stocking knitting, as it fatigues her too much. We have seen charming children's petticoats made by her, but she is not able to do much work at present. Since last week, subscriptions have been received from: M. Beer, 2s.; M. G. F., 1s.; A. G. Robson, 2s.; Miss Murray, 1s.

### "THE HOSPITAL" ENDOWED BED.

We beg to remind our readers that subscriptions for the Nurse's Bed are now due. All contributions sent to the Editor, The Lodge, Porchester Square, will appear in The Hospital. A letter from Nurse Elms will be found in another column.

### Management of Consumption.

### IV.—COUGHING.

COUGHING is so universal in consumption, and so well-known as being in the majority of cases indicative of disease of the lungs, that all people with coughs, arising from whatever cause, naturally believe their chests to be affected. Certain it is that all people with consumption cough, and equally certain is it that people with no disease at all of their lungs cough, often in a most distressing manner. The cough of consumption has nothing in it to distinguish it from other coughs. It may be a dry, hacking, incessant one, attended with but little expectoration, or it may be paroxysmal, and accompanied by the ejection of large quantities of phlegm. The dry, hacking cough with little or no expectoration is most common in the early stages of consumption, whereas the latter is more often met with in the advanced stages. Apart from consumption, people with any disease of the lungs, from the ordinary case of bronchitis to that of malignant growth, one and all have cough, which may be of any description. Then again, people with anæmia dyspepsia, and those in whom the neurotic element predominates have cough which causes them to seek relief at a chest hospital.

Coughing is greatly influenced by atmospheric conditions. On a sharp, cold frosty day one may go into a ward full of consumptives and not one will be coughing; but if that same ward is visited on a damp, mild, foggy day, the change will be apparent to all, scarcely a patient but will have an attack every now and then.

The temperature also has a great effect upon coughing. Patients who, in their own homes have hardly known what it is to sleep comfortably without coughing, are surprised to find on entering a hospital that the cough ceases to trouble them. A glance at the conditions of the two places will explain this result. The room of the consumptive is kept at a temperature something like a greenhouse for tropical plants. The consumptive ward is, or should be, kept at a temperature never above 62 degrees, and there is generally a plentiful supply of fresh air. It is well to remember this, that, in addition to other evils, coughing is aggravated by close heated rooms, and that the most rational and efficient way of treating the cough is to keep the temperature of the ward between the limits so often laid down in these papers.

However unpleasant it is to be always coughing, however disturbing to others, it must yet be remembered that coughing is by no means an unmixed evil to the consumptive; on the contrary, it is of much use to him. Coughing is an effort of nature to expel from the air passages something that is irritating them. By coughing only can the phlegm be expelled, which in consumption consists of tubercle bacilli, secretion of the bronchial tubes, and broken-down lung tissue which has become dead, and by remaining, there is only acting as a poisonous irritant. Consequently it is right and proper that patients should be encouraged to cough. Some patients persistently swallow the expectoration, and this is nearly always the case in children. The danger of this practice should be pointed out. The stomach is upset by it, and want of appetite increases, and patients render themselves liable to contract tuberculosis of the intestine, with its attendant diarrhœa, and their prospect of life is considerably curtailed. Coughing, then, should not be checked by drugs, for so long as the cough continues, that is proof positive that there is something in the air passages needing expulsion. A patient should rather be taught how to cough efficiently. He should be told when a fit comes on, to sit up in bed and fix his shoulders, and bring all the power to bear he can on the chest by contraction of the abdominal muscles. Unfortunately there exists among nurses on night duty a habit of quieting patients with cough with frequent doses of the various cough mixtures. Most of them contain squills, morphia, tinct. camph. co., and glycerine. They have a soothing effect upon the cough, are pleasant to take, and the result is they are often asked for by the patient on account of these properties, and not always withheld by the nurse. This tincture drinking grows upon the patient; he wants more every night, till at last unheard of quantities are given. But the cough is only allayed for a time; when not within reach of tincture it is as bad as ever, and in the wake of this habit follows a train of evil consequences. The very constituents of the cough mixture are just those which, when taken in large quantities, produce dyspepsia, vomiting, nausea, and a total want of appetite. The morphia and tinct. camph. co., instead of loosening the phlegm, and so aiding its expulsion, make it tighter, and take away that sensation of something irritating, which impels us to make an effort to get rid of the expectoration. It was once my unhappy lot to have evidence of a pint of cough mixture, that is, 160 doses, supplied in one night to fifteen coughing consumptives. Treating cough with sedative mixtures is just as bad and unscientific as covering up a wounded artery with rags to hide the sight of blood. In the case of the cough, the expectoration and the disease producing it is there just as much as the hæmorrhage from the wounded artery. A consumptive will persist that it is only the cough; if he could stop that he would be quite well. Consequently he is frequently supplied with medicine, the object of which is to stop the cough and delude the patient with the idea he is getting well. It should be a rule then never to give any tincture or cough mixture to a patient unless it has been specially ordered by the medical man, and even then to be as sparing with it as possible.

In nursing a patient with cough one should remember that an extra pillow will sometimes make all the difference in the world. Instead of cough mixture a little warm milk may be given, or equal parts of milk, barley water, and glycerine. This latter on repeated trials has proved very efficient. If all efforts prove of no avail, then the doctor should take it in hand, and if he prescribes sedatives, he will probably unite with them expectorants. These facts appear trivial, but attention to all, helps to make the attendant skilled in nursing chest disease.

### Parochial Nursing in Glasgow.

*(From a Glasgow Correspondent.)*

WITHIN the last few years great changes have occurred in the system of nursing at the parish hospitals of the Metropolis of the West—the City, the Barony, and Govan. It is not so long ago since the nursing of the patients in the above hospitals was entrusted entirely to the pauper inmates, whose only qualification for the position was, in most cases, their inability to perform the other work generally allotted to female inmates. It was no uncommon sight in a sick ward where there were two or three, sometimes more, cases of acute pneumonia to find the nurse stretched before the fire asleep, or half-dazed with drink, the poultices on the poor patients cold and sour, not having been changed for six or eight hours, and the bed-clothes quite damp with the perspiration from the sufferers; now, happily, there is a change for the better.

The first to move in the right direction was the Barony Board, then followed the Govan, and last of all the City Board. In both the Barony and City Hospitals the training of nurses is a special feature. In those hospitals the probationary nurses have to serve for a term of two years, and attend a course of lectures on medical and surgical nursing by the medical officers, the ward-training being entrusted to the Lady Superintendent. At the end of their term the probationers are subjected to an examination in the different subjects, and if found capable they receive certificates. In Govan, so far as we are aware, lectures have not yet been commenced, probably because there are only two probationers.

If the City Board were the last to adopt the system of trained nursing, they have been the first to move in another important direction. Up to the present, by the rules of the Board of Supervision, the governor of a poorhouse has been monarch of all he surveys; not only has he engaged and dismissed officials, but he has exercised the same authority over the nursing staff. There is no doubt that a great deal can be said in support of this system in a small poorhouse with only one or two trained nurses and no resident medical officer or lady superintendent, but where there is a large staff of nurses and a resident medical officer, together with a lady superintendent, it is entirely out of the sphere of a non-professional man to hold such authority. If the medical officers have the training of the nurses, then they ought to have authority over them. Who is best able to judge of the ability and professional qualities of a nurse? Surely not an outsider, who does not know the first principles of hospital discipline. The consequence has been that breaches of discipline and neglect of duty that would not have been tolerated in any hospital in the county have been passed over in spite of the remonstrances of those best able to judge. As a result of this, the House Committee have passed, almost unanimously we believe, a new code of rules, giving the entire charge of the nursing department to the Medical Superintendent and Lady Superintendent. This is as it should be, and is following out the custom of the majority of workhouse infirmaries in England. The great remedy for all asperities that arise when lay authority takes the place due to professional jurisdiction is the complete separation of the hospitals from the poorhouses, as is done with the great majority of unions in England, where the new system has already worked well.

### The London Hospital, Whitechapel.

The quarterly and special general Court of Governors of the London Hospital, held on the 1st inst., was largely attended. Amongst those present were Lord Sandhurst, Mr. T. H. Buxton (treasurer), Messrs. A. Ruggles Brice, T. Woollard, A. Hanbury, Maurice Abrahams, Fowell Buxton, A. F. Charrington, Spencer Charrington, M.P., S. Charrington, jun., W. M. Campbell, Bedford Fenwick, Robert Hunter, Yatman, Munro Scott, Lindley, Coope, and E. G. Tagg; Colonel Darley, Colonel Trench, Father Gorman, Father Wainwright, Miss R. Paget, Miss Busk, Miss Lindley, Mrs. Ind, Mrs. J. Coope, Mrs. Sansom, the chairman (Mr. John Hale), and others.

Great regret was expressed at the unexpected death of Dr. James Anderson, of whom all spoke most cordially, Mr. Munro Scott saying "a better man, a more noble gentleman," never entered the college and hospital. A variety of business was transacted, and an announcement made regarding the decision of the committee to grant an allowance of 2s. 6d. per week to each member of the Nursing Staff for laundry expenses. This, we may remark, is a step which the matron has for years been anxious to see carried out. The reading of the report and minutes was followed by a general discussion, in the course of which Dr. Bedford Fenwick referred lengthily to the private nursing staff which he wished to reorganize, saying that he had an intimate acquaintance of long standing with the London Hospital.

Lord Sandhurst warmly congratulated the committee on their excellent report, and spoke of the grand work done by this "admirable hospital." He said that good as committees might be they were not perfect, and he considered this was the time of year in which hospital managers were specially liable to be brought to book. When confidence was placed in a committee things should be left in their hands, and if they were put into power they should be trusted. He knew from his own experience that there were not nearly enough questions asked at these meetings by independent governors, and the public who felt aggrieved did not as they ought to do, invariably and promptly make their complaints known to the hospital authorities concerned. He objected to people going "grumbling about the town." It was much better to make a straightforward complaint to the proper people, supported by evidence, and then the grievances could be sifted and satisfactorily disposed of in the best interests of everybody. He spoke regretfully of the deficit of £21,000, but said he was never afraid of spending money for a general hospital like that of the London Hospital. While so good a record of work done can be offered, he wished it to be distinctly understood that his influence and interest would be exerted to the utmost in support of the Quinquennial Maintenance Fund Appeal.

Mr. Hale, the Chairman of the Hospital, spoke at length on the question of the numbers and work of the private nurses, which last he showed to be excellent. He further explained that Miss Fisher's pamphlets were sown broadcast after a fair inquiry into her case had been made before the House Committee. Miss Fisher had been permitted to call Sisters as witnesses, with the result that her case became considerably worse after their evidence was taken. Her second pamphlet was delivered to certain favoured governors just before the meeting, it having been kept back until after the House Committee had sat on Tuesday. It appeared to be compiled from absolutely unreliable statements, which had appeared in a nursing print. An explanation of the roof episode, of which so much has been made, was given by Mr. Hale, to the effect that when Miss Fisher was asked why she had not obtained permission to absent herself on the occasion in question, she replied, "She didn't feel inclined." (Laughter.) He was of opinion that this was the keynote of this probationer's career, and probably "she didn't feel inclined" for any of the duties belonging to a nurse's training. The pamphlets referred to were sent to some members of the resident staff, as well as to selected members of the Court of Governors, who, however, did not seem properly to value this delicate attention.

Father Gorman explained that the inquiry in the Fisher case occupied the committee for two hours, and that it was a most impartial inquiry, as a friend of the probationer in question had acknowledged.

After the transaction of some formal business, the proceedings, which were harmonious throughout, closed with a cordial vote of thanks to the chairman.

### Everybody's Opinion.

*[Correspondence on all subjects is invited, but we cannot in any way be responsible for the opinions expressed by our correspondents. No communications can be entertained if the name and address of the correspondent is not given, or unless one side of the paper only be written on.]*

### DYING—NOT DRUNKEN.

Nurse Jane says: Perhaps others will write to The Hospital, and do it better than I can; but I venture to add my plea. Miss Balgarnie wrote a letter in the *Daily News* of February 25th, which nurses must heartily sympathize with. Surely some of the ladies who make such a fuss over Indian lepers and imaginary cholera might look nearer home. It seems to me very awful that a dying woman could be neglected and locked up in a police cell—in England. It is equally dreadful to hear of workhouse patients dying at nights, and yet night nurses are thought unnecessary. There are missions to all countries, but it seems to me that charity

(including nursing) might begin at home amongst our own suffering but not picturesque heathen.

### NURSES' HOLIDAYS.

MISS CAMPBELL writes from Winchelsea: I shall be glad to receive any nurses requiring change and rest during March and April. The weather here is generally pleasant during those months, and visitors derive great benefit from a stay in our quiet neighbourhood. I would take nurses at 18s. per week, or two friends sharing the same room at 15s. each, and their comforts would be well looked after. Being a nurse myself, I understand that holidays should be restful.

### CARBOLIZED SHEETS.

"PRIVATE NURSE" writes: A good plan in cases of scarlet fever is to use two carbolized sheets, the second being a great help in preventing the small scales of skin during desquamation from flying through or under the door; two also remain moist much longer than one. To secure thorough dampness without much dripping, dip the sheets, one at a time, into a receptacle, kept standing outside for the purpose, containing carbolic $\frac{1}{20}$, and wring them out as dry as possible. Three stout nails should be put over the top of the door, and three strong tape loops sewn on each sheet. It is a quick and easy task, once in every eight or nine hours, to soak the sheets.

"AN OLD NURSING SISTER" writes: Seeing some thoughtful remarks on the nursing of scarlet fever in THE HOSPITAL, I beg to offer a suggestion with regard to the sheet. Try a piece of canvas stretched on frame the size of the door, with light hinges. It could be brushed with a mop kept in carbolic, the room to be mopped over with same, especially under bed. A piece of wood let into window, top or bottom, so that window may be open in the middle, night and day, as it keeps room sweet. Sulphur candles do all that is required to disinfect room after. Have food always before you go into a sick room. One of the reasons young nurses take fever is that they are not careful, and they forget to eat enough food. Sleep and exercise are both required, and also, no nurse ought to keep on duty sixteen hours at a time.

NOTE.—We invited suggestions as to methods for keeping a sheet wet without the constant use of mop or syringe. We shall be glad to insert any practical hints which are expressed concisely.

### "THE HOSPITAL" ENDOWED BED.

"NURSE F. L. E." writes: May I again call the attention of nurses to the proposal to establish the "endowed bed" in perpetuity (*see* THE HOSPITAL, January 14th) by means of one thousand nurses each promising to subscribe or collect 30s. or £1 and upwards within the next six months. The "bed," as most of your readers know, is for the use of nurses needing rest and change after either illness, or lengthened arduous work. Up to this date very few names have been received, and only a fortnight remains to receive them, as March 14th has been fixed to count the promises. It is needless to say unless a sufficient number take it up, the scheme cannot be realised, nor need nurses to be reminded that the sum so collected (£1,500) will enable the committee to provide board and lodging in a comfortable private house by the seaside for some tired fellow-worker every two weeks for ever! To those who prefer to subscribe every year, the "bed," as at present supported is in need of such subscription; there is work enough for both. As your correspondent, "Nurse J. R. D.," truly says, "What is one bed? We want one N., S., E., and W." Still, although we cannot command endowments at will from all points, let us secure one at least in the South.

### Cholera and Common Sense.

GOOD nurses are wanted, as THE HOSPITAL pointed out recently, for workhouse infirmaries. They are also urgently demanded for small-pox and fever hospitals. All these lie so near at hand that they might well be called "home duties." Perhaps that partly accounts for their proving less attractive than an unfamiliar disease like cholera, for everybody knows that heroines are wont to leave the unromantic present for the idealised future. But common sense still holds pre-eminence with our wisest women, and these must include many who are good nurses It has been said and written that anyone who can nurse typhoid well can nurse cholera. Those women who have worked through an epidemic of the last are comparatively few, but they all seem agreed on this one point. Applications and queries constantly reach us requesting some specific for turning inexperienced persons into cholera nurses. But the only reliable specific for this is common sense, and that should show intelligent women that the only satisfactory cholera nurses will be those who have proved successful in the nursing of other diseases. Typhoid and other fevers have often been well attended by untrained friends of the patients, but they freely own that education in general nursing would have benefited the sick persons and their anxious relatives too. A surgeon does not attempt major operations without experience of minor ones. Even the excellent "plain cook" learns the elements of her art before attempting to serve up a complete dinner. Our friend "Common Sense" shows us the why and the wherefore of this. People who wish to be entrusted with the care of cholera patients, if such ever come within their reach, had better take the lesson to heart and learn as much preliminary general nursing as they can.

### The Nurse Dolls.

THE Nurse Dolls whose travels have been reported in THE HOSPITAL from time to time, have recently received an invitation to make their appearance in public again. This time they are asked to assist at a bazaar, and to lend their passive aid in increasing the funds needed for the establishment of a cottage hospital. The dolls do not appear unwilling to go, and doubtless even feel flattered at the prospect of serving a good object; but the Editor considers them quite unfit to pay a country visit at present. They inhabit cases fitted with glass doors, but London smuts and London dust have penetrated, and each month has added a deeper shade to our poor little nurses' garments. In fact, to use the plainest of English words, the dolls are dirty! They form a large family, as many of our readers know, and the groups of uniforms are of considerable interest, and it seems a great pity the little creatures should be permanently "out of work." Soap and water, needles and thread, ingenuity and a little patience, would soon restore their original attractions. Will our readers volunteer to help in this piece of work? If several parties of friends would each arrange to renovate a certain number of the dolls, we should soon be in a position to again lend out the collection to further worthy charitable objects. Suggestions and offers of help can be sent to the Editor, The Lodge, Porchester Square. This appeal has already received some very kind responses, and we shall be pleased to distribute our little nurse dolls to the friends willing to undertake their toilettes when arrangements have been made for all of them.

### The History of Nursing.

Notes of an introductory lecture given by Dr. Shaw to the nurses of the Bristol Royal Infirmary.

(*Concluded from page* cliv.)

A.D. 1529.—At the time of the dissolution of the monasteries by King Henry VIII. there were three hospitals in London—St. Bartholomew's, St. Thomas's, and Bethlehem. These, which were nursed by nursing orders, like the monasteries were compulsorily closed. In the thirteenth year of the King's reign, the Lord Mayor, Aldermen, and Common Council presented a petition to the King for the re-opening of the hospitals. Petition asks for provision for "the ayde and comforte of the poore sykke, blind, aged, and impotent persones, beying not hable to helpe themselves, not havynge anye place certeyn whereyn they may be lodged, cherrysshed and refreshed, tyll they be cured and holpen of theyr dyseases and syknesse." Hospitals were re-opened under lay administration, and apparently were attended by "physicians, surgeons, and appotecarys" mentioned in the petition aforesaid, but the nursing does not seem to be alluded to.

A.D. 1557.—In the "Order of the Hospitalls of K. Henry VIIIth and K. Edward the VIth, [viz. St. Bartholomew's, Christ's, Bridewell, St. Thomas]" published "by the Maior Cominaltie and citizens of London, Governours of the Possessions, Revenues and Goods of the sayd Hospitalls" in this last year of the reign of Queen Mary, the rules for the "nurses and keepers of wards" indicate that women of a comparatively low class were now engaged as nurses in place of the male and female members of the nursing orders who discharged this duty prior to the Reformation.

From the beginning of the sixteenth century all over Europe the Church was no longer able to provide for the growing needs of sick nursing, and hospital building became again the function of the laity, when for nearly a thousand years there had been no sick nursing outside the walls of cloisters or hospitals of the Orders. In Italy rich nobles, in England merchants, in Germany princes, built hospitals. Guy, a London bookseller, founded the hospital called after him in 1721.

In connection with the Roman Catholic Church many associations for nursing the sick have been formed since the year 1500. Various male societies, called "Brothers of Mercy," and a still larger number of societies for women called "Sisters of Mercy." The most important of these latter are the Sisters of St. Vincent, founded in 1617 by the Parisian ecclesiastic Vincent de Paul, and the Borromean Sisters, founded in 1625 at Nancy by a lawyer, who left his fortune to found a female nursing society.

The Protestant Churches made no attempt at public nursing until the 19th century.

A.D. 1829.—Elizabeth Fry gives instruction to the nurses at Guy's Hospital, having been previously in correspondence with the poet Southey about the insufficient and inadequate attention paid to the inmates of hospitals as well as in workhouses. This seems to be the first instance in this country of instruction being systematically given to nurses.

1836.—Pastor Fliedner instituted, at Kaiserwerth, on the Rhine, a Deaconesses' Institute, for the training of women to be nurses; the institution was founded and established, as nearly as possible, upon the pattern of the early Christian Church; the deaconesses wear a veil, but do not take vows, as in the Roman Catholic nursing orders. The modern system of training nurses dates from the establishment of this institution.

1854 5.—Miss Florence Nightingale, who had been trained at Kaiserwerth, with other nurses, went to the seat of the Crimean war to nurse the wounded soldiers. She was the first well-known "lady nurse" in England since the time of the Reformation. On her return to England a public subscription of money, amounting to £45,600 was made, to be presented to her as a testimonial, which, however, she declined to accept for herself.

1858.—Mrs. Wardroper, Matron of St. Thomas's Hospital, relieves the nurses of menial duties, and generally ameliorates their position.

1859.—District nursing commenced in England.

1860.—The money collected as a testimonial to Miss F. Nightingale, called the "Nightingale Fund," was placed in the hands of trustees in order to endow a training school for nurses, supervised by Miss Nightingale herself. Owing to the nursing reforms which had already been introduced at St. Thomas's Hospital, arrangements were made with Governors of that institution, and the training school was opened there in June of that year.

1864.—The Secular (Genevese) International Society of the Red Cross, founded for nursing the sick and wounded in the time of war.

1887.—English Knights of St. John revived for the establishment of ambulance classes, giving instruction in "first aid" to the wounded.

Staff Nurse. Old Style.

The greater masters of the common-place,
Rembrandt and good Sir Walter—only these
Could paint her all to you; experienced ease
And antique liveliness, and ponderous grace,
The sweet old roses of her sunken face;
The depth and malice of her sly grey eyes,
The broad Scot's tongue that flatters, scolds, defies
The thick Scot's wit that fells you like a mace.
These thirty years has she been nursing here,
Some of them under Syme, her hero still.
Much is she worth, and even more is made of her,
Patients and students hold her very dear.
The doctors love her, tease her, use her skill,
They say "The Chief" himself is half afraid of her.

Staff Nurse. New Style.

Blue-eyed and bright of face, but waning fast
Into the sere of virginal decay,
I view her as she enters day by day,
As a sweet sunset almost overpast.
Kindly and calm, patrician to the last,
Superbly falls her gown of sober grey,
And on her chignon's elegant array
The plainest cap is somehow touched with caste.
She talks Beethoven; frowns disapprobation
At Balzac's name, sighs it at "poor George Sands."
Knows that she has exceeding pretty hands;
Speaks Latin with a right accentuation;
And gives at need (as one who understands)
Draught, counsel, diagnosis, exhortation.

—W. E. Henley, *Edinburgh Royal Infirmary*, 1874.

Bibliography.—Rupprecht's "Die Krankenpflege," Puschman's "History of Medical Education," "The Speaker's Commentary," Hobart's "Medical Language of St. Luke," "New Testament Commentary for English Readers," Holy Bible (Revised Version and Authorised Version), "The Order of the Hospitals, &c.," 1557, "Training Schools for Nurses," New York, 1883, Anderson's "Medical Nursing," "A Book of Verses," by W. E. Henley, 1888, Pliny's "Natural History," translated by Philemon Holland, &c., &c.

### In Memoriam.

Dr. James Anderson, of Wimpole Street, who died on Tuesday, February 28th, after only a few hours' illness, will be missed by a very large circle of friends. Nurses now working in all parts of the world have kindly memories of the helpful sympathy which was ever at the service of rich and poor, young and old. Dr. Anderson's years of work as one of the out-patient physicians at the London Hospital brought him in contact with a vast number of the sick poor, and he was as well-known and well-beloved in the great East-end as he was amongst his West-end patients. As lecturer to the nurses at the London Hospital Dr. Anderson was also most popular, and he often spoke of that teaching as congenial work. Of Dr. Anderson's appointments and successes others will write, but in the "Nursing Supplement" it is well to speak specially of his attitude towards nurses and probationers, by whom his invariable sympathy and kindly interest will be terribly missed. Other men may earn equal fame, but no one can ever gain more affection than surrounded one whom we all most deeply lament.

### Minor Appointments.

WILTSHIRE COUNTY COUNCIL.—Miss T. H. Aitken has been appointed Lecturer on Sick Nursing and Domestic Hygiene for the Wiltshire County Council. Miss Aitken was trained at the Royal Southern Hospital, Liverpool, and has had charge of a medical ward in the Royal Hospital for Sick Children, Glasgow, and afterwards of the children's wards at the Bolton Infirmary and Dispensary. Miss Aitken has also had valuable experience in district nursing, and for the last year has been acting as Assistant Superintendent at the Royal Infirmary, Liverpool.

HULL DISTRICT NURSE.—Miss Jane Reilly has been appointed District Nurse by the Hull District Nursing Association. Miss Reilly was trained at Guy's Hospital, where she remained for four years, then she joined the Q.V. Nursing Institute at Wolverhampton for one year, the Royal Infirmary, Derby, for thirteen months, and afterwards the Kent Nursing Institute, West Malling, for thirteen months. Such valuable and varied experience renders Miss Reilly specially qualified for her new work, in which we heartily wish her success.

### Notes and Queries.

### SPECIAL NOTICE.

The contents of the Editor's Letter-box have now reached such unwieldy proportions that it has become necessary to establish a hard and fast ruleregarding Answers to Correspondents. In future, all questions requiring replies will continue to be answered in this column without any fee. If an answer is required by letter, a fee of half-a-crown must be enclosed with the note containing the enquiry. We are always pleased to help our numerous correspondents to the fullest extent, and we can trust them to sympathise in the overwhelming amount of writing which makes the new rules a necessity.

### Queries.

(56) *Training.*—What does the Oakley System consist of? What are the Church Army nurses?—*Janetta.*

(57) *Tables.*—Kindly tell me if there is a small book called "Posological and Therapeutical Tables?" I have been told so, buthave asked in vain at several booksellers.—*Sphinx.*

(58) *Midwifery.*—Where can I get six months' training to obtain certificate as midwife.—*Nurse Jessie.*

(59) *Smoking.*—A matron will be glad to know if it is customary to allow patients as a general rule to smoke in bed?—*Matron*

(60) *Flannelette.*—Where can the specially woven flannelette be procured which was described in account of "Plaster of Paris Jacket," page 317. Feb. 11th.—*Sister.*

(61) *Probationer.*—Where can I find out particulars as to nurse training schools?—*Helen.*

(62) *Holidays.*—I want to know what time off duty I should get at a general hospital.—*Enquirer.*

(63) *Insanity.*—Is there any simple book on the nursing of the insane published for nurses?—*A Country Nurse.*

(64) *Cottage Nursing.*—Useful book required.—*Nurse Eva.*

(65) *L.O.S.*—Information wanted.—*District Nurse.*

### Answers.

(56) *Training* (*Janetta*).—Write for particulars to Miss Broadwood, of Reigate; also to Church Army Training Branch, Edgware Road.

(57) *Tables* (*Sphinx*).—You should write to either Lewis, Gower Street, or Kimpton, Holborn—both are medical booksellers. There are several books such as you speak of. Student's Pocket Prescriber, by Aubrey Husbands, 1s. Craig's Posological Tables, 1s. Doses of British Pharmacopia. 4d. Syllabus of Materia Medica, by Alex. Harvey and Alaxander Dyce Davidson, 1s. 2d.

(58) *Midwifery* (*Nurse Jessie*).—Any lying-in hospital, such as City of London, in City Road, Queen Charlotte's Hospital, British Lying-in, Endell Street, Maternity Hospital, Jeffries Road, Clapham, General Lying-in Hospital, Lambeth, or St. Mary's Home, Plaistow. If you write to the matrons you will get all particulars as to fees, examinations, &c. If you are a trained nurse you could get free midwifery training through the Workhouse Infirmary Nursing Association, 6, Adam Street, Strand. The hon. secretary will give you particulars.

(59) *Smoking* (*Matron*).—Certainly not, except, for instance, during Christmas festivities. We have heard of patients infringing the rules against smoking in bed in institutions where untrained attendants are employed. It could not be tolerated in general hospitals, except for patients able to leave the wards and enjoy smoking in the garden or in a pavilion set apart for the purpose, and in the Seaman's Hospital, Greenwich, it is allowed after 5 p.m. In any difficulty arising at a cottage hospital the authority of the medical man would always be given in support of the matron.

(60) *Flannelette* (*Sister*).

(61) *Probationer* (*Helen*).—You had better get "How to Become a Nurse and How to Succeed," by Honnor Morten, Scientific Press, 140, Strand. Price 2s. 6d.

(62) *Holidays* (*Enquirer*).—You should put this question in writing to the Matron of the hospital where you are going to be trained. The hours vary in different institutions.

(63) *Insanity* (*A Country Nurse*).—Yes. "Mental Nursing" by Dr. Harding. Published by Scientific Press. Price

(64) *Cottage Nursing* (*Nurse Eva*).—Mrs. Dacre Craven's book, "District Nursing," is a useful one.

(65) *L.O.S.* (*District Nurse*).—See answers, Nos. 58 and 61, this week.

### For Reading to the Sick.

### BURDENS.

MANY of us can take up a heavy burden and carry it some little way, but if we were condemned to walk with it ten or twenty miles every day for a month together, we should break down under the load. We might contrive to stagger on a little while, but our strength would fail us before long, and if we did not drop it the load would crush us. It is necessary for us to have some one to share it with us, to encourage us in bearing it bravely; indeed, to take the whole weight on his own shoulders if it can be. So it is with sickness; we can go on a little while in our own strength, but as day succeeds day, and our pains do not lessen, we feel that it is pressing down our souls just as a great weight presses down the body. I think if it were a load that made our backs bend unduly we should not allow it to crush us, we should very soon cry out for help to those near us; and yet in our sufferings of body and mind we murmur and are discontented, our hearts are filled with bitterness because we do not turn to One who has promised and is always ready to bear our griefs and carry our sorrows. Christ helps us in so many ways. He puts love and sympathy into the hearts of our friends and attendants, and by their care and kindness our trials become easier. Grateful we should be to them, but our hearts should rise higher in blessing and praise to Him who is the author of all these good things. Our Heavenly Father never puts on us more than we can bear, although we are ever ready to think so. Faith and trust in His wisdom and love will cool the parched tongue and soothe the throbbing head. As our days so shall our strength be is a promise which Christ is daily fulfilling.

We may ask why these heavy loads are sent? The answer is not far to find; it is because God would get at our souls through our bodies. Our hearts are so hard or so set on our worldly enjoyments that we want something startling to bring us to a sense of our dependence on a higher power. There is no happiness so complete as putting ourselves entirely into God's hands, to give up struggling against His will, to lie still and feel that His strength is sufficient for us to bear the heat and burden of the day and to cast all our care upon Him, for He careth for us, is the height of wisdom and of bliss. In the watches of the night Jesus will bend o'er us and give us strength to cope with our pains by filling our minds with thoughts of peace and hope.

"He giveth rest, more perfect, pure, and true,
While we His burthen bear;
* * * * *
Take as His gift the pain, the gift brings yet
A truer happiness."

### Hints to Nurses.

THE "Dorcas Thimble," made by Ch. Horner, of Halifax, Yorkshire, ought to be appreciated by all nurses, for it is one which will wear for an indefinite time without showing signs of those holes which are so trying to busy people. The "Dorcas" is not only a pretty silver thimble, but a very strong, serviceable one.

### London Obstetrical Society.

NURSES CHAWNER AND RIDAL, of the Fir Vale Union Hospital, Sheffield, have passed the L.O.S. examination.

### Wants and Workers.

We have to thank Mr. Gofton Salmond for his kind offer of a surgical letter for the poor woman in whose behalf we appealed last week to our readers.

### Some Australian Experiences.

*(Concluded from page clxviii.)*

### MY RESIGNATION.

I must not forget to mention my "little Norah," as she was generally called. Such a good servant, and much interested in nursing, she was of the greatest assistance to me, and delighted in it, but she was the only help I had, the second servant was always a trouble. Sunstroke and D. T. cases gave us much anxiety. They get "a touch of the sun," as they call it. Once a man I had to nurse was so violent that I had to get another to watch him. He was a bit of a character, he combined so many "callings." He was town crier and bellman, clog dancer and low comedian, a bit of a "digger." A better man to watch I never had, for go when I would to see how they were, "Bob" was always wide awake. I was quite sorry when he left the "field," for later on when a "poison case" came in and I got a man to watch, whenever I went in he was fast asleep with the patient watching him.

As the winter wore away I got more goats, and as my starved nannie had a little kid we had quite a small family. I had chickens, but the hawks, snakes, and iguanas took them away as soon as they were hatched; it was very disheartening.

During the cool weather we had several very nice pic-nics to a place called the Springs. There is a real spring and such a beautiful gorge close by, which in the wet season is a big waterfall, so we used to go out in a large party about sixteen or eighteen, take our food and spend a most pleasant day. It is about fourteen miles from the town, so some rode and others drove. Upon one of these occasions the party was so large we had a coach; then a lady and two gentlemen drove tandem in a high dogcart, following that, a buggy and pair of cream ponies, the rest on horseback; and very lovely it is to get a good scamper through the Bush. Considering the distance we were up country we got very good mounts, and there never was a *fête* of any kind that I was not included in. I fear it spoils one for town life afterwards, Bush life is so free.

We also had races midwinter and Christmas; the latter I did not attend, it being my busy time; but the former I could. Of course, it is a very small affair, indeed. Some of the "squatters" come in to them, and the whole thing is more a social gathering than anything. The Masonic Ball was the great annual event, and weeks beforehand the "Masons" work very hard at emblems, mottoes, and decorations of every description, and on the night everyone puts on his "war paint." I went to two of them, and it would have surprised many a town person to have peeped in and seen how very nicely everything was done. This takes place in June, so that it is really cool for dancing. The Masonic Hall is a fine building, and, when nicely waxed, makes a capital ball-room; the dresses upon each occasion were of the best description.

One thing greatly startled me when I first went to live in this hot region, and that was the quick manner in which the dead were put underground. I had to learn by a terrible experience the great importance and necessity for it. It happened thus: the first death which took place was a poor woman whose husband worked in a "claim" seven miles outside the town. Being anxious that he should see his wife again, in the evening of the day on which she died I rode out to his camp to tell him. He arrived at the hospital early next morning, but in that short time the corpse presented a most ghastly spectacle. I never kept another so long again—it was too terrible. I shudder now at the recollection of it.

There are a good many blacks about the "Gulf country." A large number camped a short distance from the hospital, so, under the protection of two trackers and a policeman, I visited the camp and saw them in their natural state; their "Gunyahs" are very stuffy and small, but the curious framework they erect, and sleep on the top, with fires lighted round to make a good smoke, to keep off the mosquitoes, is to all appearance most uncomfortable. One would think the smoke would choke them, but they do not seem to mind. They are a harmless, childish sort of creature when under the "eye of the law," but I question whether they would be so a few miles from the town; they are treacherous at all times. I used to take a walk in the early morning near the camp, until someone warned me against it, telling me they might have "Matron Pie" some morning for breakfast.

A lot of work can be got out of them for a little tobacco and any old clothing, which they always put on the wrong way. A heavy penalty is attached to giving them spirits, and no black fellow is allowed about the township after sunset; drink sends them completely mad, then trouble arises.

Sometimes the Normanton blacks came up, then there was an awful to-do. Some were invariably killed, and the yells and wailing were things to be remembered, as all their dogs joined in the chorus, the town dogs took it up, and then the "dingos" in the "bush."

Directly at the back of the hospital was dense "bush." It was quite easy to be lost in a very few minutes. One young fellow, a stranger, was lost for three days, and was found only about a mile away. There was also a poor man, subject to fits, whose wife missed him on one of the race days, and although the police, trackers, doctor, and several others were out searching, he was not found for five hours, then only about seven minutes' walk from the back of our paddock. It was supposed he had a fit and died from exposure to the heat. This danger made the nursing of D.T. and sunstroke cases most anxious work, I was so afraid of them getting away.

Thus passed my first year, when I had serious thoughts of resigning, but I had formed friendships up in this desert region which made the hardships easier to bear, so resolved to try another, a step I deeply regretted afterwards.

I had ventured to hope that, as the drought had broken up the year before, and the wet season been exceptionally severe, there might be a milder one this time. I was mistaken, the summer came in furiously hot, and by Christmas, not only was the fever very bad, but my good little handmaiden also fell ill, and a very hard time again fell to my lot, more than a repetition of the previous summer. I scarcely know now how I got through it, but the result was a complete breakdown of my own health—fever, "Gulf lumps," with suppuration and general debility.

I could not lay up—there was no one to take my place; so I simply struggled through, constantly breaking the abscesses under my arms by carrying heavy weights and making beds, so at length I resigned, glad to rest on my oars, and, after spending a most delightful holiday with my dear friends, decided to take a trip to England to visit old scenes and friends before taking up my work again in Sydney or Melbourne.

Such was my seven years' Colonial experience; and although the latter part was the roughest, I would not have missed it, on account of the kind friends I met. But I would strongly advise any nurse never to take a distant position without first securing travelling expenses both ways, as £20 for the same out of £100 is a large sum, when other expenses are so heavy, and such hard work so impairs one's health, and only a rest does one any good.

A word about "gold miners." When you get the genuine article they are generally kindly and grateful, but the class that grumble is very much mingled with them, and although I nursed a very large number during my ten months, I did not meet so very many of the jolly, good-natured, happy-go-lucky "diggers" that one reads about. Of course, the community is mixed, but the large majority are Irish, next come Cornishmen, Scotchmen, a few Germans, Greeks, Italians, and I only met with one Frenchman.

So far as my work is concerned, I shall never regret having had to rough it, on account of the extremely good "cases" I had, which, humanly speaking, could not have recovered but for the skilled nursing bestowed; but I question whether it is right to send nurses to these far-away spots single-handed. I have tried to show the great kindness I received during my seven years' sojourn in Australia, and I look forward to my return to the land of my adoption with great pleasure, for before long I hope to go out and work once more with the kindly people from whom I borrow the name with which I sign myself. Cornstalk.

### The Book World for Women and Nurses.

[We invite Correspondence, Criticism, Enquiries, and Notes on Books likely to interest Women and Nurses. Address, Editor, THE HOSPITAL (Nurses' Book World), 428, Strand, W.C.]

### TO OUR READERS.

WITH the view of meeting the wishes of a large circle of readers, we have determined to devote a special section of the "Nursing Mirror Supplement" to books and literary matters especially calculated to interest women and nurses. We are aware that, to very many, a careful classification of the book advertisements proves most helpful, and we shall endeavour to so arrange as to enable every reader of THE HOSPITAL to have information concerning every new book found of value. We shall be glad to give insertion to inquiries concerning rare, or out of the way books which may have a special interest of their own. We shall also welcome the co-operation of our readers to the extent of directing our attention to any British, Foreign, Colonial, or American books calculated to prove of service to women and nurses. Publishers will please direct all books intended for review to the Editor, 428, Strand, W.C.

### NEW BOOKS.

"Hygiene of the Sick Room," * by William Buckingham Caufield, A.M., M.D., is a book which will interest every nurse who is fortunate enough to meet with it. Although many pages can be chiefly commended to qualified nurses, yet there are others which affect all heads of households and persons interested in sanitation. Such a passage as the following is of general, not only individual, application. "In using disinfection, it should be borne in mind that that method which is the most thorough, which disinfects the object most quickly, is the one to be preferred. Again, all things being equal, the simplest, cheapest, and most harmless method of disinfection should be employed. Lastly, disinfection should be carried out by those thoroughly skilled in the work." The list of disinfectants recommended is a long one, and the chapter on tuberculosis is excellent, and so is the one on Asiatic Cholera. The little volume is not only an instructive but a very readable one, and many nurses will be glad to rub up their hospital knowledge by reference to these pages. We do not quite see the use of a piece of muslin, a foot square, being hung in the room, even if it is soaked in disinfectant, but the New York Board of Health puts the recommendation amongst their other regulations. Great stress is laid upon the care of babies' eyes, and the prevention of ophthalmia neonatorum. Ventilation and food are well treated, and in connection with the latter the nourishing properties of chocolate receive due attention. As regards the notification of infectious diseases we learn that this lies in the hands of either the doctor or householder and that "city health boards are not governed by the most recent knowledge is shown by the fact that the Health Board of Baltimore requires notification of such a disease as mumps, but does not require an inspection in cases of typhoid fever."

* "Hygiene of the Sick Room," by WILLIAM BUCKINGHAM CAUFIELD, A.M., M.D. (P. Blackiston, Son, and Co., 1012, Walnut Street, Philadelphia.)

### THE BOOK WORLD FOR WOMEN AND NURSES—*continued.*

"Handbook of Massage" † by Emil Kleen, M.D., Ph. D, translated from the Swedish by Dr. Hartwell, is a work calculated to give students a true idea of an agent applicable to many, but certainly not to all diseases. Dr. Weir Mitchell has written a short introduction to the American edition, in which he speaks of the book as a "calmly scientific statement of the uses and effects of massage." The translator, Dr. Hartwell, has also added a preface and an excellent index to this useful volume. Besides careful and elaborate description of cases for which properly carried out massage is beneficial, Dr. Kleen gives very clear directions (chap. IV.) regarding the diseases in which it is contra-indicated, and this chapter will be particularly valuable to the student. Chap. XII. is an exhaustive treatise on diseases of the eye and of the technique of massage of this organ. The book will be useful for study as well as reference by persons interested in massage, and it will certainly considerably enlarge the reader's views of the art by showing the endless varieties existing in the treatment, and also the numbers of obscure diseases, such as *neuroses of the ear*, for which, on Pollitzer's authority, it is beneficial.

† "Handbook of Massage," by EMIL KLEEN, M.A. (P. Blackiston, Son and Co., 1012, Walnut Street, Philadelphia.)

